# Valorisation of algal biomass to value-added metabolites: emerging trends and opportunities

**DOI:** 10.1007/s11101-022-09805-4

**Published:** 2022-03-02

**Authors:** V. S. Uma, Zeba Usmani, Minaxi Sharma, Deepti Diwan, Monika Sharma, Miao Guo, Maria G. Tuohy, Charalampos Makatsoris, Xiaobin Zhao, Vijay Kumar Thakur, Vijai Kumar Gupta

**Affiliations:** 1 Radiological and Environmental Safety Group, Department of Atomic Energy, Indira Gandhi Centre for Atomic Research (IGCAR), Govt of India, Kalpakkam, Tamil Nadu India; 2 Department of Applied Biology, University of Science and Technology, Meghalaya, 793101 India; 3 School of Medicine, Washington University, Saint Louis, MO USA; 4 Department of Botany, Sri Avadh Raj Singh Smarak Degree College, Gonda, UP India; 5 Department of Engineering, Faculty of Natural and Mathematical Sciences, King’s College, Strand Campus, The Strand London, London, WC2R 2LS UK; 6 Molecular Glycobiotechnology Group, Biochemistry, School of Natural Sciences, Ryan Institute and MaREI, National University of Ireland, H91 TK33 Galway, Ireland; 7 Future Business Cambridge, Cambond Limited, Centre Kings Hedges Road, Cambridge, CB4 2HY UK; 8 Biorefining and Advanced Materials Research Center, Scotland’s Rural College (SRUC), Kings Buildings, West Mains Road, EH9 3JG Edinburgh, UK; 9 School of Engineering, University of Petroleum & Energy Studies (UPES), 248007 Dehradun, India; 10 Center for Safe and Improved Food, Scotland’s Rural College (SRUC), Kings Buildings, West Mains Road, Edinburgh, EH9 3JG UK

**Keywords:** Algal biomass, Biorefinery, Algal biomass valorisation, Algal metabolites, Value added products, Circular bioeconomy

## Abstract

Algal biomass is a promising feedstock for sustainable production of a range of value-added compounds and products including food, feed, fuel. To further augment the commercial value of algal metabolites, efficient valorization methods and biorefining channels are essential. Algal extracts are ideal sources of biotechnologically viable compounds loaded with anti-microbial, anti-oxidative, anti-inflammatory, anti-cancerous and several therapeutic and restorative properties. Emerging technologies in biomass valorisation tend to reduce the significant cost burden in large scale operations precisely associated with the pre-treatment, downstream processing and waste management processes. In order to enhance the economic feasibility of algal products in the global market, comprehensive extraction of multi-algal product biorefinery is envisaged as an assuring strategy. Algal biorefinery has inspired the technologists with novel prospectives especially in waste recovery, carbon concentration/sequestration and complete utilisation of the value-added products in a sustainable closed-loop methodology. This review critically examines the latest trends in the algal biomass valorisation and the expansive feedstock potentials in a biorefinery perspective. The recent scope dynamics of algal biomass utilisation such as bio-surfactants, oleochemicals, bio-stimulants and carbon mitigation have also been discussed. The existing challenges in algal biomass valorisation, current knowledge gaps and bottlenecks towards commercialisation of algal technologies are discussed. This review is a comprehensive presentation of the road map of algal biomass valorisation techniques towards biorefinery technology. The global market view of the algal products, future research directions and emerging opportunities are reviewed.

## Introduction

Algae are versatile multicellular ubiquitous organisms known for their biotechnological and environmental significance. Algal forms are either micro or macroalgae and are known to be the oldest living microbes of the earth, which also explains its vast diversity (Stern et al. 2010). The existence of these mighty microbes dates back to 3.5 billion years (Margulis 1981). Algal species are known to subsist in coastal and aquatic habitats; however, they are also reported in extreme conditions such as hot springs, polar regimes, salt pans etc. (Show et al. 2017). Algae are photosynthetic organisms, thus having the ability to convert solar energy and carbon dioxide into biomass and oxygen. They catalyze the dehydration of HCO_3_^−^ using carbonic anhydrase as compensatory mechanism for CO_2_. The marine algae mostly assimilate CO_2_ by the Calvin–Benson cycle (C3), however some species utilize Hatch–Slack cycle to enhance the photosynthetic process (Liu et al. 2021). These oxygenic photosynthetic microbes are diverse in their morphology ranging from unicellular (prokaryotic cyanobacteria) to multicellular (multicellular eukaryotic algae) and their length vary between 0.2 μm and 65 cm existing in about 50,000 algal species (Christaki et al. 2011).

In modern days, immense research attention has been thrown on algae as they are considered a potential feedstock for food, feed, fuel and several other value-added metabolites. Specific products obtained from algal biomass such as PUFA, essential oils, vitamins, antioxidants, carotenoids and other metabolites are reported to possess remarkable dietary and therapeutic applications (Sathasivam et al. 2019). Algae are largely exploited naturally available food supplements as their cellular composition meets human dietary needs (Koyande et al. 2019a). Despite being a rich source of protein, amino and fatty acids that are ideal for human consumption, aqua and poultry feeds etc., commercial cultivation of algae, more specifically the microalgae began in recent decades, and it still needs enormous research attention (Borowitzka 2018). From an industrial viewpoint, algae used for waste remediation integrated CO_2_ sequestration to reduce the carbon footprint is an environmentally viable valorisation method. Daneshvar et al. (2022) discussed novel carbon capture techniques and valorisation of carbon sequestered biomass for production of biofuels, oleochemicals and other value added products. Cultivation of algae has an edge over the terrestrial feedstocks as they have higher biomass productivity, higher photosynthetic efficiency, cultivable round the year, and do not require arable land with minimal nutrient inputs (Tan et al. 2020a).

Suitably designed and optimised biorefineries for productivity, energy use and cost for algal biomass valorisation represent a sustainable route to maximise its commercial utilisation (Kumar and Singh 2019). Biomass valorisation determines quantitative and qualitative extraction of value-added metabolites besides making the process cost effective and energy efficient (Gonzalez-Lopez et al. 2020). In this direction, the present review aims to explore the emerging trends and the novel technologies in algal biomass valorisation for extraction of viable products.

## Feed stock potential of algal biomass: an overview

Algae are envisioned as promising candidates in the renewable energy market as third generation biofuels besides being ideal agents for natural supplements, medicines and feeds. As per the global algae market analysis report 2019–2025, algae market is expected to grow by US$414.8 Thousand, guided by a compounded growth of 6.6%. Several microalgae are used to capture atmospheric CO_2_ with unique carbon concentrating mechanisms and are considered a viable option for greenhouse gas mitigation (Khan et al. 2018). Algal species have formed carbon concentrating mechanisms and can establish themselves in complex waste and pollutants making them lucrative bio-factories for biorefinery approaches (Singh and Dhar 2019). Recent studies reported algal biomass could uptake harmful emission such as SO_x_, NO_x_ and convert them into valuable by-products (Singh and Thakar 2015). Gendy and El-Temtamy (2013) reported that a kg of algal biomass can sequester 1.83 kg of CO_2_ coupled with effective uptake of other harmful flue gases and nutrient load from complex waste waters.

Bioenergy potentials of algal biomass is the current topic of global research as algal fuels are efficient, fast growing and yield high quality fuel without distressing the food crops. Algal fuels include both liquid fuels (biodiesel, bioethanol) and gaseous fuels (biomethane, biogas) and are renowned in global energy markets as a mighty replacement to conventional fuels (Zabochnicka-Świątek 2010). An increasing demand for algae-based bioethanol and biodiesel as fuel alternatives has been observed in the recent years. Microalgae *Chlorococcum infusionum, C. reinhardtii UTEX 90, C. vulgaris and Chlamydomonas reinhardtii* are model candidates for large scale bioethanol production that research has identified (Daroch et al. 2013). Macroalgae also possess competent yields and globally exploited for bioethanol. Species of *Gracilaria*, *Undaria* and *Laminaria* have been investigated for bioethanol production via biorefinery routes (Tan et al. 2020b).

Algae are an affluent source of bioactive compounds namely the proteins, amino acids, carotenoids, lipids, fatty acids of dietary value, polysaccharides, auxins etc. Recent studies have identified anti-bacterial, antioxidant, anti-inflammatory, antitumor and antiviral properties, in addition to the established phenomenon that algal biomass itself acts as dietary and nutrient source of food and feed standards (Michalak and Chojnacka 2015). Besides, microalgal and macroalgal metabolites are high value compounds that are used as cosmetics and nutraceuticals. Algal extracts are exploited as anti-ageing compounds, sunscreen agents, moisturisers, emulsifiers etc. (Aslam et al. 2021). Pimental et al. (2018) discussed on the existing commercial cosmetic products from algae namely the carrageenan, Gelcarin^®^ PC 379, alginate etc., and highlighted that these compounds have been recognised as functional skin care products.

**Fig. 1:**
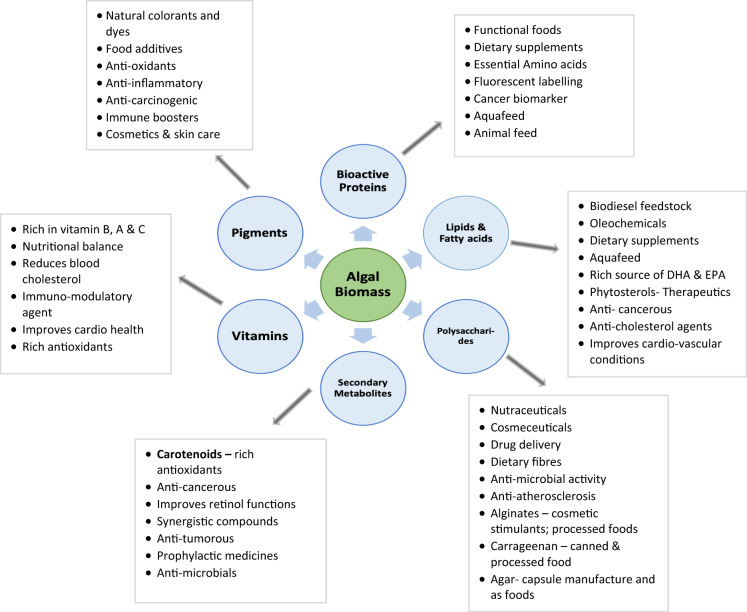
Feedstock potentials of algal metabolites and its biotechnological applications

Nutritional value of the algal compounds holds notable industrial value capable of replacing the animal protein and lipid sources in the aquaculture industry (Geada et al. 2021). Though, algal carotenoid is a secondary pigment, yet it possesses therapeutic attributes. Of the different class of carotenoid compounds that exist, β-carotene and astaxanthin have enormous applications as anti-cancer, anti-inflammatory agents and are used to treat metabolic disorders, gastric ulcers etc. (Sathasivam et al. 2019). Antioxidant properties of *Pterocladia* sp., *Porphyra* sp., *Fucus nesiculosus* and *Padina gymnospora* have been well documented. The remarkable credentials of algal biomass upsurged its value in global market which parallelly demanded intensive research on cultivation and valorisation methods to attain a sustainable supply chain (Fleita et al. 2015). Figure 1 depicts the feedstock potentials of algal metabolites and its diverse biotechnological applications for value product recovery.

## Value-added metabolites from microalgae

### Bioactive proteins

**Fig. 2:**
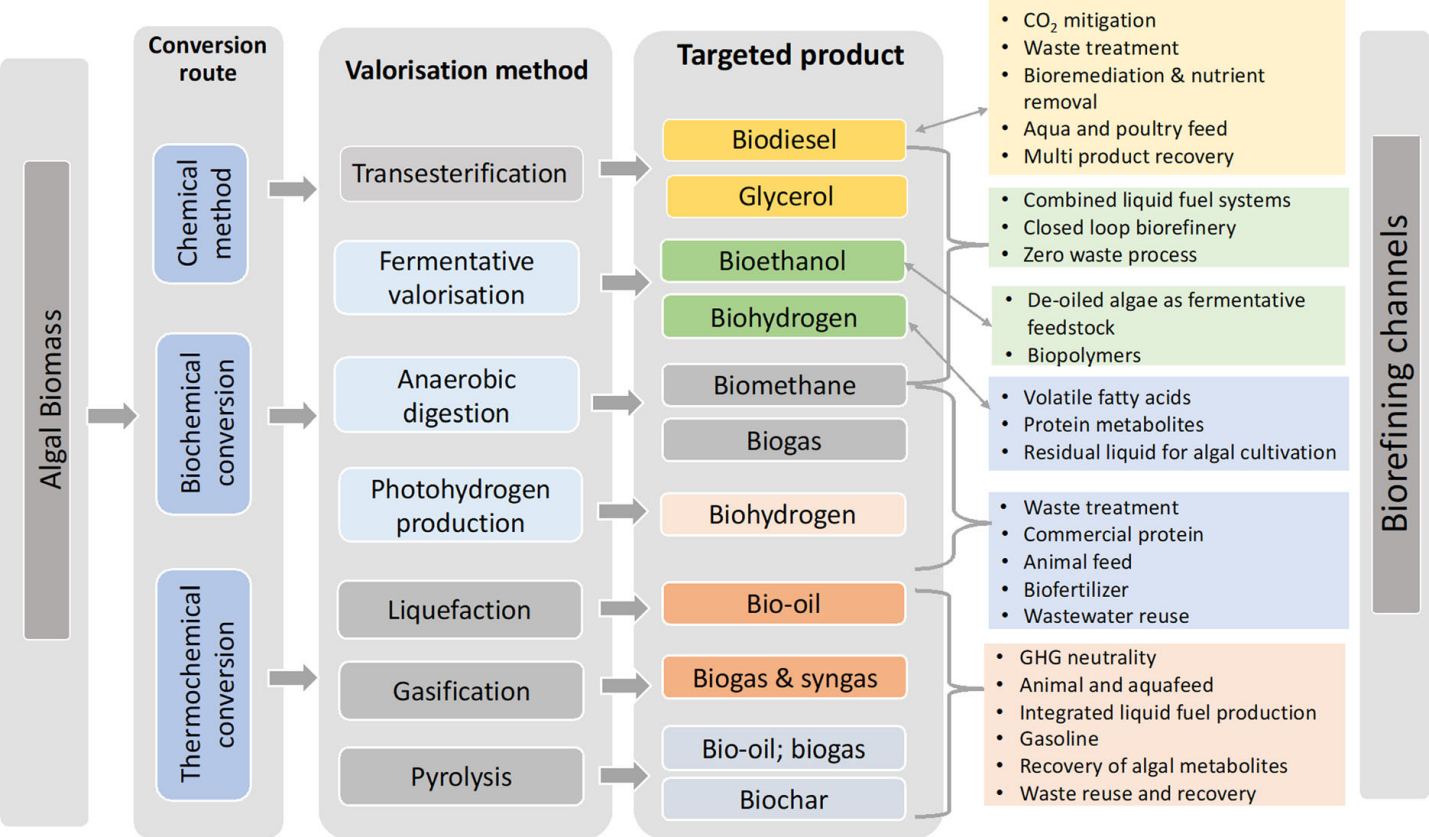
Biomass valorisation and its plausible biorefinery channels

Algal proteins and amino acids have garnered attention over the last few decades. The biomass contains about 40–60% of proteins with significant properties making them desirable food and feed replacements (Suganya et al. 2016) (Fig. 2). Food and Agriculture Organization (FAO) adjudged the dietary significance of algal proteins and stated that they have the edge over the “basic conventional food items”, which also makes them highly competitive in food and feed market (Becker 2007). Compared to macroalgae, microalgae are highly explored due to its protein and amino acid profile with high dietary value. Microalgal species *Aphanizomenon *sp., *Chlorella *sp., *Dunaliella* sp. and *Arthrospira* sp., with a high protein content of 50% and above are utilised for human consumption (Koyande et al. 2019a). While other microalgae, namely *Tetraselmis* sp., *Nannochloropsis* sp., *Dunaliella* sp., and *Chlamydomonas* sp., have shown efficacy as an animal and aquaculture feed (Hashmein et al. 2019; Sousa et al. 2008).

Selected cyanobacteria, *Spirulina* sp. (45–65%), *Arthrospira maxima* (70–75%), *Synechococcus* sp. (60–65%), have also gained research interest due to its rich amino acid composition (Chronakis et al. 2011). Irrespective of the morphotype, algae are an affluent source of essential amino acids including lysine, valine, leucine and tryptophan. In a study conducted by Mišurcová et al. (2014), the RDI (Recommended Daily Intake) of different algal species were compared. Lysine, Leucine, Alanine, Glutathione and Asparagine were dominant in the amino acid pool of selected algae. The RDI of commercially used algae as a source of EAA (essential amino acid composition) were 3 g (*Chlorella pyrenoidosa*), 13 g for *Spirulina platensis* and *Spirulina pacifica* respectively. Pereira et al. (2018) experimented on different protein extraction methods and their impact on the protein fractions’ quality and quantity to be used as functional foods in *Spirulina* sp. LEB18. Protein and amino acid concentrates from several *Spirulina* sp. have been graded as GRAS (Generally Recognised as Safe) by FDA (Food and Drug Administration). Chen et al. (2019) investigated the functional properties of three microalgal species. The study highlighted the importance of the biomass valorisation methods as it could significantly impact the properties of the algal proteins. The vast biodiversity of algae and its habitat distribution is attributed to the substantial differences in its protein and essential amino acid composition (EAA); hence a high throughput screening of a large number of species would enable the identification of novel species with remarkable nutritive value (Dawczynski et al. 2007).

### Polysaccharides

Marine algal polysaccharides (MAP) have immense potential for industrially viable and novel products. They are used in global markets as nutraceuticals, cosmeceuticals, pharmaceuticals, for drug delivery, fertilisers and as aquafeeds (Rajendran 2020). In recent years, MAP’s have placed their foot in newer areas of research namely the bioremediation, carbon sequestration and in genetic engineering technology (de Jesus Raposo et al. 2013). Macroalgal polysaccharides, including the fucoidan, ulvan, rhammnan sulfate, and laminarin sulfate of seaweed origin, have found their application as therapeutic agents for atherosclerosis (Patil et al. 2018). Lipid reducing and anti-coagulant properties of laminarin sulfate extracted from brown alga laminaria and carrageenan from red algae are delved into meticulously for commercial applications (Smith et al. 2018; Necas and Bartosikova 2013). Sulphated polysaccharides from microalgae and cyanobacteria show antiviral activity against HIV-1, HSV-1, Flu-A (de Jesus Raposo et al. 2013). Agar, which is the combination of two different polysaccharides agarose and agaropectin extracted from *Gelidium* sp. and *Gracilaria* sp. is globally used as texturing agent in foods, while agarose a non-sulphated form of agarose, is used extensively in protein and molecular research (Vavilala and D’Souza 2015).

The complex structural configuration of the MAP is a bottleneck for its sustainable production and its commercial value. The molecular composition of the MAP vary across the algal genera, hence requires a precise valorisation method (Pierre et al. 2019). Jönsson et al. (2020) critically reviewed the impact of the diverse valorisation methods on the quantitative and qualitative profile of the MAP. Enzymatic modification is acclaimed as a plausible method to recover MAP without compromising its intrinsic properties. However, the cost efficacy of the enzymatic valorisation process can be improved by aiding on technologically viable platforms such as the combine biomass processing, biorefining etc. Gaignard et al. (2019) detailed the compositional heterogeneity of the algal polysaccharides among the tremendously diverse algal species. Hence, a consensus should arrive on the extraction and purification method specific for algae or the type of MAP in order to widen the commercializing aspect. Total biomass valorisation in a biorefinery approach may be sorted to extract MAP as exopolysaccharides are comprehensively utilised for biomedical applications (Yen et al. 2013).

### Lipids, fatty acids and essential oils

Microalgae are a promising source of lipid and fats. Algal lipids are considered a potent third-generation biofuel feedstock; however, they are interesting candidates for dietary fats and supplements and could potentially be the equivalent synthetic products (Kumar et al. 2020b). The vast and varied habitat distribution of algae implicates the diverse cellular lipid composition in these organisms (Uma and Dineshbabu 2020). Microalgae are sustainable sources of essential oils rich in long chain n-3 PUFA (Poly Unsaturated Fatty Acid), omega-3 fatty acids, DHA and EPA along with moderate levels of MUFA (Monounsaturated Fatty Acids) and lesser amounts of SFA (Saturated Fatty Acids) (Remize et al. 2021). The global market size of omega-3 fatty acids in 2017 accounted to be 2.49 billion and is projected to increase by 7% by 2027 (Omega 3 Report 2020). The surging exigence for the essential fats coupled with the unsustainable availability of conventional sources, algal PUFA and oleochemicals have gained attention as novel alternatives (Harwood 2019). Reports of Batista et al. (2013) and Borowitzka (2013) reported the high value microalgae for PUFA are *Chlorella* sp.,* Nannochloropsis* sp., *Odontella aurita*, *Parietochloris incise*, *Phaedactylum tricornutum*, *Porphyridium cruentum*, *Diacronema vlkianum*, *Isochrysis galbana*, *Schizochytrium* sp. *Spirulina platensis* and *Ulkenia* sp. Another lucrative class of lipids from algal biomass are the sterols, which primarily includes phytosterol, ergosterol, fucosterol and cholesterol. Algal phytosterols are used as therapeutic steroids, predominantly as anti-cancer and anti-cholesterol agents (Francavilla et al. 2012).

The dietary value of the algal lipids and the bio-accessibility of the long chain PUFA have been studied in copious interventional trials (Cottin et al. 2011). The findings suggest that about 50–100% of algal DHA and EPA are made available to humans and have significantly improved the cardiovascular conditions. DHASCO-T and DHASCO-S are commercial DHA capsule from *Schizochytrium *sp. delivered competent levels of nutritive fats in humans (Lane et al. 2014). The bioequivalence algal oils both as capsules (DHASCO) or as fortified foods had a positive effect on the plasma phospholipid and erythrocyte DHA and performed better compared to the synthetic supplements (Arterburn et al. 2007).

Algal biomasses tend to present distinctive bio-accumulation profiles and growth dynamics with the changing environment or in the presence of stressors. Hence, optimisation approach is essential to extract the key metabolites without compromising its dietary value (Laurens et al. 2017). For pilot scale production of algal lipids and fats, it is essential to establish an in-depth understanding of algal genomes, biochemical pathways, and organism response to environmental, nutrient stress, and genetic manipulation (Wells et al. 2017; Makatsoris et al. 2019). *Phaeodactylum tricornutum* with about 30% omega-3 fatty acid is an industrially claimed algae for human consumption. Hamilton et al. (2015), overexpressed the heterologous genes responsible for PUFA production that enhanced the yield by 7.5% with an elevated EPA productivity of 43.13 mg/L in reduced light conditions. Despite the availability of multiple valorisation methods for algal lipids, fermentative technology has been the optimistic method for consumable DHA and EHA (Barclay et al. 2013). *Schyzochitrium* sp. and *Ulkenia* sp. were successfully cultured in heterotrophic carbon from valorised by-products that resulted in enhanced DHA content (Gupta et al. 2012). Oliver et al. (2020) reviewed the cultivation strategies of PUFA form algal biomass and suggested that heterotrophic cultivation of algae is cost burdening and unsustainable for DHA production using renewable carbon sources (sugarcane waste, molasses, lignocellulosic biomass) could be a bio-economic approach. Interestingly, algae are commercially acclaimed as “super foods” and are consumed as whole food (dried biomass) for their dietary and therapeutical benefits. *Arthrospira* sp. and *Chlorella* sp. are consumed as a dried powder, marketed for its valuable fatty acid rich in γ-linolenic acid and essential fatty acids. Algal super foods are reported to have anti-tumour and anti-coagulant activities besides acting as a nutrient buffer in the human system (Liu and Hu 2013; Martinez et al. 2018).

### Vitamins

Algae have been in human diet for over decades and are a pivotal source of nutrients in aquatic food web. The biomass potential of algae for its protein, fatty acid and pigment is well established, however recently the vitamin load gathered momentum of research (Buono et al. 2014). Owing to the limited availability of vitamins in vegan diets, algal foods are considered excellent source of folate, vitamin B12 and niacin (Watanabe et al. 2013). The largely cultured algae for the purpose of commercial vitamin-B production are *Chlorella pyrenoidosa, Arthrospira platensis* and *Spirulina* sp. Studies of Edelman et al. (2019) affirmed that *Chlorella* are a rich source of bioactive-folate with rich concentrations of 5-HCO-H4 folate and 5,10-CH^+^-H4 folate. However, the study also highlighted the significance of the extraction methods on the quantity and quality of the recovered folate. Additionally, biomass harvesting, drying, and processing methods may also impact the vitamin’s quantity (Jeske et al. 2011).

Watanable et al. (2013) reported the bioavailability of vitamins that are few folds higher in *Chlorella* sp. while the bioactive pool of the *Spirulina* sp. are rich in pseudo-vitamin which makes them less ideal for therapeutic applications. Lima et al. (2018), postulated that B12 binding of the microalgal vitamins in humans could be enhanced by expressing the human B12-binding protein intrinsic factor (IF) in their model organism *Chlamydomonas reinhardtii*. In another report by Andrade et al. (2018) microalgae *Spirulina* and *Chlorella* are found to be excellent source of vitamin B (Thiamine-B1, riboflavin-B2, pantothenic acid (B5), folate and cobalamin-B12) and vitamin A (β-carotene). Algal vitamins have proven health benefits in human metabolism. Vitamin A from microalgae accelerates the immune function and cellular interaction, while vitamin B3 has been instrumental in cholesterol metabolism, reduces blood glucose and improves cardio-vascular conditions (Solomons 2012). A recent study has identified a marine diatom *Skeletonema marinoi* that is rich in ascorbic acid and could accumulate large quantities of vitamin C. Being a water-soluble vitamin, it functions as an immuno-modulatory agent. Also, it aids in collagen bio-synthesis (Smerilli et al. 2017).

Microalgae have been identified as reserves of vitamin E and are synthesised from *Chaetoceros calcitrans, Nannochloropsis oculate* and *Dunaliella tertiolecta* for biotechnological applications (Santiago-Morales et al. 2018). The specific bioavailable concentration and functional roles of different vitamin rich microalgal bio-factories have been reviewed by Del Mondo et al. (2020). The authors highlighted the technical setbacks and the conundrum that lay ahead in the road map of sustainable vitamin production from algal biomass. Koyande et al. (2019a), critically reviewed the microalgal species that are exploited as a vitamin resource since 1990 and identified the research gaps that exists in development of viable algal vitamin food and feed. The development of algal food capable of delivering fat-soluble vitamin in human system needs better insights to identify industrially competent valorisation methods. Such comprehensive research information on extraction and delivery systems are sparsely available and demands research attention (Ferraces-Casais et al. 2012).

### Secondary metabolites

Algae are a potential feedstock for its versatile secondary metabolites synthesised at a later stage of growth (Fig. 1). Both fresh water and marine algae accumulate large secondary metabolites capacities, including carotenoids, sterols, polyphenols, etc. (Leflaive and Ten-Hage 2007).

#### Carotenoids

Carotenoids, the light harvesting pigments are present in aquatic food chain in abundant quantities. Algae, rich in carotenoids and β- carotene are exploited for commercial production owing to its rich antioxidant and anti-cancerous properties (Guedes et al. 2011; Takhashi et al. 2015). The other carotenoid derivatives with health benefits are astaxanthin, fucoxanthin and zeaxanthin, all of which are known for scavenging harmful oxygen with appreciable antioxidant values (Galasso et al. 2019). The presence of one or more of these pigments is species specific. The algae that are mainly known for its carotenoid abundance are, *Dunaliella salina, Nanochloropsis* sp., *Spirulina platensis, Tetraselmis suecica, Dunaliella tertiolecta* and *Asterarcys quadricellulare* (Singh et al. 2019). The health benefits of the algal carotenoids are abundant; they act as synergistic compounds in preventing cellular damage in cancer patients, repress cardiovascular disorders, reduce cholesterol in blood streams, used as antioxidants and are aidful in retinol functions (Galasso et al. 2019).

#### Polyphenols

Polyphenols are an interesting class of compounds with multiple health benefits. Algal polyphenols include phenolic acids, iso-flavonoids, flavonoids, coumarins, lignins, and phenolic polymers (Manach et al. 2004). The vast therapeutic and health attributes of polyphenols include its antioxidant, anti-microbial, and anti-tumoral, anti-cancer, anti-diabetic, anti-cancer properties that have intrigued the biotechnologist globally (Gastineau et al. 2014). In the macroalgal group, brown algae and seaweed are immensely rich in polyphenols (Fernando et al. 2016). Profound antioxidant property and inhibitory effects of four economic macroalgae were associated with the bio-activity of the phenolics (Yuan et al. 2018). Microalgae on the other hand, possess polyphenols amongst their diverse genera. Higher concentration of polyphenolic acid and flavonoids were reported in *Spirogyra* sp., *Euglena* sp., *Caespitella pascheri, Nostoc* sp., *Nodularia spumigena* and *Arthrospira* sp. (Jerez-Martel et al. 2017). Jimenez-Lopez et al. (2021) reviewed the health benefits of polyphenols and discussed the pros and cons of different extraction methods. The study suggested that algal polyphenolics are not only to be used as therapeutics, but they could be also utilised as prophylactic and palliative care.

### Oleochemicals

Microalgae are plausible feed stock for the value added oleochemicals as these oleaginous algae are a rich source of lipid and fats that contain ~ 70% oil by weight (Chisti 2007). Oleochemicals are a class of chemical compounds obtained from fats, lipid and fatty acids and find its application in alternative energy, biosurfactants, lubricants and others (Yu et al. 2014).

#### Biodiesel

Algal oleochemicals offer sustainable environmental solutions, including green energy (biodiesel) integrated with CO_2_ sequestration and waste minimisation in a biorefinery approach (Wang et al. 2020). Lipid being a low value compound, is effectively channelled for producing a by-product or a process such as bioremediation, carbon mitigation, while recovery of biodiesel is the primary target. These biotechnologically viable compounds are flexible to genetic and metabolic engineering and can be modified for enhanced performance (Pfleger et al. 2015). Oleochemicals from algal lipids have gained momentum in recent years, as algae are classic chassis framework species for evolution and adaptation and are collaborative towards nutrient optimisation, strain development, and genetic manipulation (Marella et al. 2018). Biodiesel, the liquid fuel from algal lipids is valorised through different conversion technologies namely the transesterification and thermochemical conversion. Being a carbon neutral fuel, algal biodiesel can significantly replace conventional fuel, and the biomass can be utilised to quench harmful greenhouse gases from the atmosphere (Chye et al. 2018). *Chlorella *sp. are the most commercially used algae for biodiesel production and have been genetically modified for enhanced lipid yield and carbon capture (Mata et al. 2010; Medipally et al. 2015). Table 1 shows the variable lipid content of algal genera and its diverse biotechnological potentials. Bioenergy supply chains could be improvised by the emerging technological platforms, where one or more forms of energy could be processed from the feedstock biomass in a closed loop (Gupta et al. 2014). By this way, algal fuels will become potential candidate in the energy sector with high biorefining values.

**Table 1:** Variable lipid content of algal genera and its biotechnological applications

No.	Name of the algae	Total Lipid yield (dry wt)	Biotechnological application	References
1	*Chlorella sorokiniana DSR*	Up to 83%	Biofuels and bioremediation	Hena et al. (2015)
2	*Schizochytrium* sp.	Up to 80%	Dietary lipids and biofuels	Sajjadi et al. (2018)
3	*Botryococcus braunii*	Up to 75%	Hydrocarbons	Kleinart and Griehl (2021)
4	*Schizochytrium* sp.	50–77%	Biofuels and supplements	Schlagermannet et al. (2012)
5	*Botryococcus braunii*	25–75%	Biofuels	Schlagermannet et al. (2012)
6	*Nannochloropsis* sp.	36–68%	Biofuels and aquafeeds	Schlagermannet et al. (2012)
7	*Nannochloropsis* sp.	45%	CO_2_ mitigation, commercial production of pigments and lipids	Thawechai et al. (2016)
8	*Nitzschia* sp.	45–50%	Biofuels	Schlagermannet et al. (2012)
9	*Neochloris oleobundans*	35–45%	CO_2_ bio-fixation and biodiesel	Razzak (2018)
10	*Neochloris oleobundans*	35–74%	Biodiesel	Schlagermannet et al. (2012)
11	*Nannochloropsis* sp.	55%	Biofuels and aquafeeds	Sajjadi et al. (2018)
12	*Dunaliella* sp. *ABRIINW-11*	47%	Pharmaceutical and aquaculture applications	Gharajeh et al. (2020)
13	*Scenedesmus dimorphis*	10–40%	Biofuels	Milledge et al. (2011)
14	*Chroothece richteriana*	35% (PUFA)	Therapeutics and dietary supplements	Aboal et al. (2014)
15	*Monoraphidium contortum*	31.5%	Liquid biofuels	Bogen et al. (2013)
16	*Chlorella vulgaris*	30%	Biofuels	Shokravi et al. (2020)
17	*Chlorella variablis*	24%	Biodiesel	Nirmala ( 2020)
18	*Oscillatoria* sp.	Up to 20%	Biodiesel and biocalcification	Uma et al. (2020)
19	*Spirulina subsalsa*	19%	Biodiesel	Uma et al. (2020)
20	*Ulva* sp.	Up to 15%	Human Health foods and animal feeds	Moustafa and Batran (2014

#### Surfactants

Algal surfactants possess novel structural configuration that suggests the broad scope of its *in-situ* functional properties for diverse biotechnological applications. Algal lipids, principally the membrane lipids and the monounsaturated fatty acid derivates are ideal source of oleochemicals that are of particular interest as bio-surfactants. Fatty alcohol derivatives namely sulfates, alkoxylates, amines and ether sulfates are versatile surfactants (Pfleger et al. 2015). Algal oleochemicals derivatives are widely used in industries that produce laundry products, emulsifiers, oil industry, thickeners, foaming agents etc. Kainarbayeva et al. (2019) extracted oleochemicals from three microalgal species, *Spirulina sp, Botryococcus sp* and a wild strain of the Sugur soda lake culture and evaluated their biosurfactant properties. The study identified that ester exchange using methanol could be a plausible strategy to accelerate the surfactant accumulation in *Spirulina sp.* The investigation of Radmann et al. (2015) on the cultivation strategies of three microalgal species, spotted carbon source as the key factor that is pivotal in enhancing its surfactant properties. The crucial role of carbon in fatty acid metabolism is explainable and it opens the scope of coupling carbon sequestration with biosurfactant synthesis from lipid rich microalgae. Fatty acids of the crude algal biomass along food hydrolysates were trans esterified and subjected to epoxidation for production of plasticiser and surfactants (Pleissner et al. 2015). Compounding of two conventional methods provides immense opportunities to minimize waste and acquire industrially viable multi-products.

## Systemic exploration of recent trends in algal biomass valorisation for fuel production

Algae is a potential source for valuable products like food, feed, and biofuels for pharmaceuticals, nutraceuticals and cosmetics industries. The integrated valorization approaches help in the extraction of valuable products from algae thus expanding the horizon of bioproducts in the therapeutics and food industry. Sequential extraction of valuable compounds from the algal biorefinery results in minimization of waste, expanding revenue and full utilization of feedstocks. These valorization processes are important to utilize algae as a favourable resource in biorefinery.

### High throughput fermentative valorisation

Algae are a promising renewable feedstock for fermentation which is the productive valorisation method to maximise algal resource recovery as a range of biofuel (e.g., bioethanol) and value-added compounds. Suitable biomass pre-treatment and valorisation methods are essential for maximum hydrolysis of sugar, carbohydrates and biofuels. To fasten the process of high throughput fermentation, the pre-treatment and hydrolysis of the biomass must be combined. Novel methods of bio-hydrogen and bio-ethanol products aim to improvise the fermentation and photolysis processes (Dasgupta et al. 2010). The next generation biofuels, the 4th generation biofuels rely upon novel technological process like “Photo-fermentation” or combined algal biomass process using genetically modified microalgae for biofuel production through biorefining (Silva and Bertucco 2019). High-throughput fermentation is a novel method for bioethanol as it is a ‘zero waste’ strategy. El-Dalatony et al. (2019) subjected *Chlamydomonas mexicana* to successive fermentation process and the residual biomass was transesterified resulting in simultaneous extraction of carbohydrate, protein and lipid extraction. The study affirmed that high throughput fermentation can be implemented to generate other forms of biofuels integrated with bioethanol and can minimise cost involved and waste generation maximally. The fermentative products obtained by serialised valorisation showed similar profile to that of the intact cell extraction processes. Lai et al. (2016) demonstrated a novel bioethanol strategy the “selective fermentation. Microalga *Scenedesmus sp.* was selectively fermented using granular sludge to yield volatile fatty acids thereby retaining the other metabolites for biotechnological applications. Subhash and Mohan (2014) investigated the plausibility of utilising de-oiled algal biomass as a feedstock for biohydrogen through dark-fermentation using anaerobic mixed microflora as a catalyst. The results affirmed that algal biomass can be successfully fermented to obtain two forms of bio-energy. Xia et al. (2015), reviewed the various modes of high throughput fermentation which includes dark fermentation combined photo-fermentation, anaerobic digestion for biomethane and hydrogen generation from algal biomass. The organic remains (acetates, protein, acetate) that leaves the process following the dark fermentation can be further subjected to a photo fermentation to yield viable fatty acids. In an earlier study by Xia et al. (2013), substantial yields of biohydrogen (144.9 mL H_2_/g Vs) and biomethane (161.3 mL CH_4_/kJ/g Vs) from *Nannochloropsis oceanica* using an integrated fermentative reaction was recorded. Anaerobic fermentation of algal biomass releases substantial quantities of volatile fatty acids (VFA) as intermediary compounds, which could in turn be used for large scale cultivation of microalgae in a closed-loop attempt (Kumar et al. 2020a). A novel approach for carbon recycling from microalgal biomass and utilisation of the resultant VFA through thermophilic anaerobic fermentation was attempted by Kim et al. (2019). The integrated biomass valorisation model achieved an accelerated carbon recycling ratio of ~ 40% and fatty acid recovery of 60%. High throughput fermentation of algal biomass is an efficient strategy to valorise biomass completely and harness maximum energy cost-effectively.

### One step conversion of biomass to metabolites

Biomass valorisation for viable biotechnological products is executed in multiple steps. While biofuel production from algae employs pre-treatments methods, hydrolysis, lipid extraction, transesterification, pyrolysis purification (Fig. 2) etc., However, methods like direct biomass conversion, *in-situ* transesterification unswervingly converts the biomass into the targeted products bypassing severing pre and post treatment methods. This technological upgrade is profitably put into practice for biofuel processing. Biodiesel extraction from *Serratia *sp. revealed a one-fold increased product recovery in lesser time as compared to the conventional methods (Kumar et al. 2017). Performance efficiency of one step valorisation methods using supercritical methanol was compared with microwave assisted transesterification while the former was found to be superior in being energy efficient and yielding stable biodiesel product with no or minimal impurities (Patil et al. 2012). An integrated biomass deconstruction process was evaluated using a thermophilic bacterium *Defluviitalea phaphyphila*, capable of assimilating sugars to direct brown algal biomass to bioethanol. The fermentative capacity of the bacterium was utilised via genetic manipulation to enhance the biodiesel production capacity in algae (Ji et al. 2016). In a novel approach, biomass of *Chrococcus *sp. was simultaneously harvested and pre-treated by a fungal species *Aspergillus lentulus* in anaerobic condition. The bio flocculating fungal species notably enhanced biomethane production in *Chroococcus *sp and rapidly solubilised the sugars. One step biomass valorisation approach appears cost-effective and simplified the complex harvesting process of algal biomass (Prajapati et al. 2016).

Microwave assisted transesterification is a recent technological movement for direct conversion of whole biomass to biodiesel. Cancela et al. (2012), demonstrated a base assisted-microwave transesterification of three macro algae and observed significant reduction in reaction time (3 min) as compared to the base- catalysed transesterification method (3-5 h). Microwaves been instrumental in extracting lipid from algal biomass; however, total valorisation of algal biomass via direct transesterification has gained research attention. This avoids the arduous pre-treatment methods (Kapoore et al. 2018). One step conversion of microalgal biomass to biofuel through microwave (MW) assisted transesterification has enhanced FAME recovery in *Nannochloropsis gaditana.* MW aided biodiesel production could be carried out in bioreactors as an integrated one pot bio-process for self-sustained biodiesel production (Menéndez et al. 2014).

### Transesterification of biomass to biofuels

**Fig. 3:**
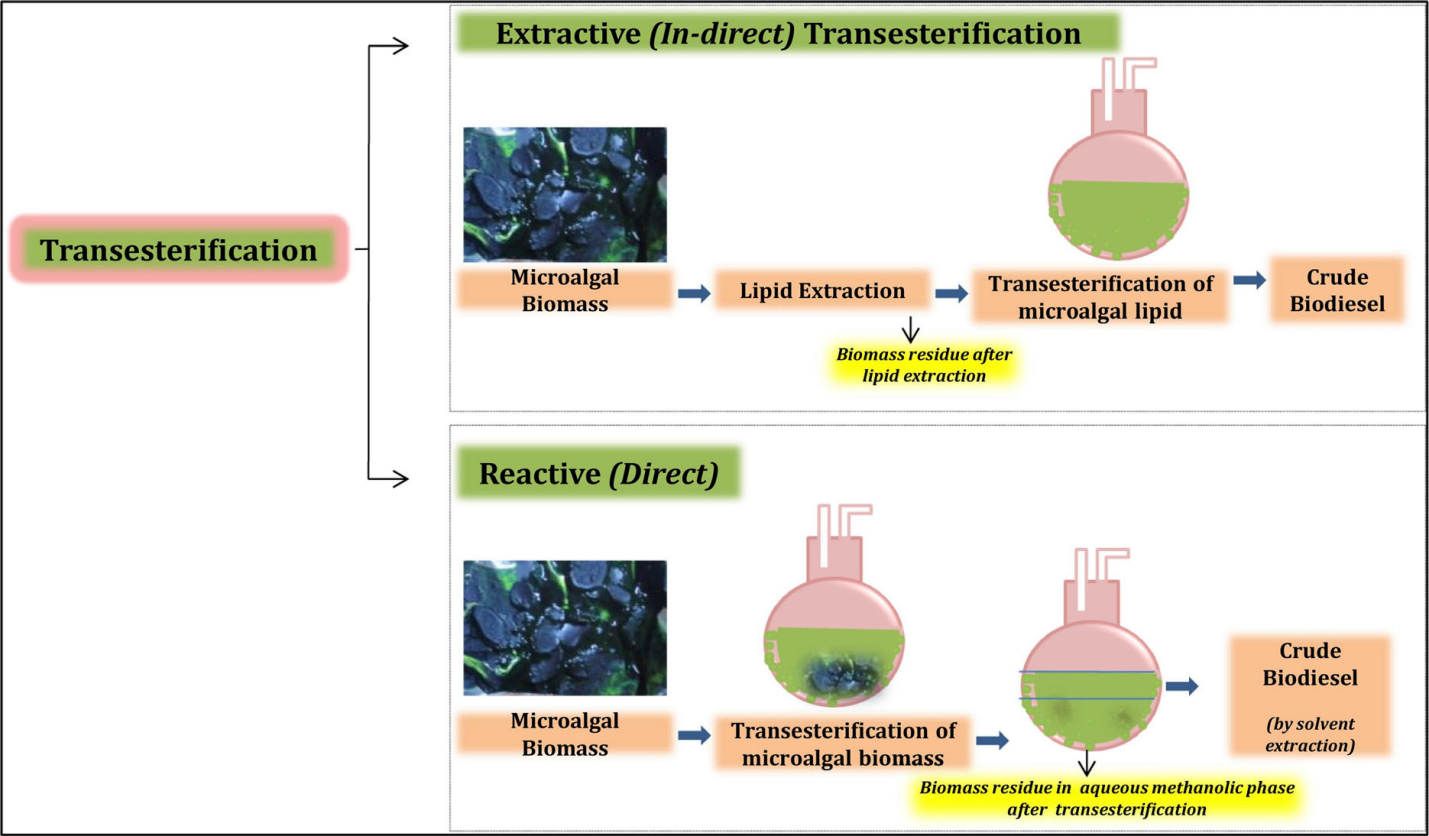
Transesterification of algal biomass (in-situ). With Permission from Elsevier. Copyright© 2021. License Number: 5107730473664 (Karpagam et al. 2021)

Energy is the ultimate face of global economy. With rapid exhaustion of fossil fuels and increasing global warming, alternative energy has gained momentum in recent years. Microalgal biodiesel is a promising third generation feedstock that are produced through transesterification and thermo-chemical routes. However, to accelerate the efficacy of the process, technology driven sophisticated biomass valorisation methods are essential (Park et al. 2015). Conventional transesterification reactions are categorised into acid, base or enzymatic catalytic methods, however recent advances such as the heterogeneous catalysis, microwave or ultrasound assisted and supercritical transesterification methods have significantly upsurged the scope of biodiesel in energy market terms appreciable cost-economy (Hossain, 2019). In-situ extractive and reactive transesterification of microalgal biomass has gained attention due its significantly lower number of process steps that avoid cost intensive steps such as drying of algal biomass and extraction of lipids (Fig. 3).

Nano-technology has ventured into biofuel research and envisioned as a novel approach for closed-loop utilisation of algal biomass. Nanocatalyst has an edge over the other catalyst in being product specific with high catalytic activity (Akubude et al. 2019). Mesoporous nano-particles can extract lipid fractions from microalgal cells without disrupting their cell wall, enhancing its opportunities for multiple applications and near total biomass utilisation as the biomass remains available for bio-processing (Malik and Sangwan, 2012). In a simple reaction, the nano-biocatalyst containing the nano particle are infused into the reaction mixture containing the alcohol and algal biomass. This process is efficient in biodiesel production and leaves the biomass uncompromised (Zhang et al. 2013). Enzyme lipase immobilised on magnetic nanoparticles is a proven technology for biodiesel from algal biomass. Nematian et al. (2020), demonstrated biodiesel production from *Chlorella vulgaris* using a lipase immobilised nanoparticle catalyst and achieved a recovery rate of 71.9% biodiesel. The study highlighted the sensitivity and recyclability of the lipase-nanoparticle catalyst with a pronounced performance efficacy to over five times (70%) against the conventional methods.

The technical complexity of the homogenous transesterification which involves multiple processing steps, slow reaction time and one time usability have been overcome using heterogenous catalyst that are available as solid forms and can be reused. Homogenous transesterification methods involve laborious purification steps and are cost burdening. Heterogenous catalyst can be reused and recovered for sustained utilisation (Faruque et al. 2020) and have a high tolerance for Free Fatty Acid (FFA) with appreciable specificity and longevity (Thangaraj et al. 2019). FAME recovery from two microalgae *Chlorella protothecoides* and *Scenedesmus obliquus* were evaluated using molybdenum oxide fixed on a mixed oxide ZrTiO heterogeneous catalyst to convert the oleic acid fractions to biodiesel specifically. The high methyl oleate content of the algal species chosen for the study revealed pronounced conversion rate of 84 and 35%, respectively. β-Strontium silicate was tested for algal oil production from *Spirulina platensis* and a maximum FAME recovery of 97.8% was achieved (Singh et al. 2020). Thus, heterogenous catalyst can profoundly valorise algal biomass on a targeted bio-fraction (Veillette et al. 2017). Given its efficient performance, newer heterogenous catalyst and transesterification methods are constantly identified for specific FAME yields.

###  Anaerobic digestion

The rich organic load of the microalgae is harnessed to generate biofuels by anaerobic digestion methods (Fig. 2). The biochemical pool of algae constructs them as a valuable anaerobic digestate to be utilised in waste management coupled bioenergy technologies (Stiles et al. 2018). Algal digestate is an immensely useful source of nutrients (Nitrogen, phosphorus and potassium) and have been feedback in a closed-loop approach for large scale algal cultivation to aid the growth of microalgae *Chlorella vulgaris* (Ras et al. 2011). This approach leaves no biomass residue while significantly shrinks the cultivation cost (Ward et al. 2014). Biodiesel from *Tetraselmis suecica* was coupled with biomethane production by anaerobic digestion of the residual biomass in the glycerol environment, resulting in an enhanced biomethanation (438.46 ± 40.50 mL) (Santos-Ballardo et al. 2015). Valorisation of algal biomass for efficient energy recovery could be technically improved using anaerobic hybrid reactor, by coupling other carbon-rich substrates and algal biomass. The complementary substrates (olive mill wastewater) to algae biomass to feed anaerobic digestion not only enhanced the bio-methanation process but surpassed the additional biomass pre-treatment methods (Assemany et al. 2018).

Anaerobic digestion (AD) integrated with hydrothermal carbonisation (HC) to convert macroalgal species *Fucus serratus* and *Saccharina latissimi* was demonstrated by Brown et al. (2020). Hydrothermal processing at higher temperatures (above 150 °C) significantly enhanced the biomethane generation and reduced the net energy demand of the reaction process. A similar effective valorisation method was developed by integrating the HC and AD in microalgal consortia, resulting in an enhanced net energy recovery from algal hydro-char and the anaerobic digestion of the residual biomass (Marin-Batista et al. 2019). Anaerobic digestion coupled hydro-pyrolysis using algal consortia (*Phormidium* sp. and *Chlorella* sp.) resulted in an enhanced (50%) bio-methanation observed in solid phase hydro-pyrolysis reaction platform. Aqueous pyrolysis accelerated the recovery of nutraceuticals compounds leave no part of the biomass as a residue. In a novel attempt by Llamas et al. (2021) microalgal consortia was used to anerobically digest complex solid waste in a non-convential 2-step valorisation process that resulted in production of short chain fatty-acids (SCFA) and biogas. The biorefining approaches via anaerobic digestion of algae signifies versatility of the biomass potentials for sustainable bioprocess (Choudhary et al. 2020).

### Photobiological hydrogen production

Hydrogen production from photosynthetic algae in oxygenic, hypoxic or anoxic conditions have been of particular interest over the last few decades. With the emerging technologies, biohydrogen from algae has attained commercialisation in several parts of the world (Eroglu and Melis, 2011). Despite being a potential renewable energy feedstock, biological H_2_ production from algal biomass suffers from limiting factors that includes the super sensitivity of the hydrogenase enzyme to O_2_ molecule, competing metabolic pathways for the photosynthetic reductants and other scaling up challenges which demands improvised technological platforms (Khosravitabar 2019). Bio-hydrogen from algae is produced via two established modes, bio-photolysis (direct and indirect) and photo fermentation. However, recent valorisation methods, namely dark fermentation, immobilisation methods, anaerobiosis, etc., have widened its scope in a commercial prospective (Rashid et al. 2013). Immobilised microalgal cells on a suitable support matrix is envisaged as an ideal choice for pilot scale production of hydrogen from algal biomass as it not only enhances the productivity but is regarded a robust method for pilot plants (Wang et al. 2018). These systems are highly resistant to oxygen sensitivity and can utilise the available light effectively (Kosourov et al. 2018).

A novel biomass valorisation for improved hydrogen using oxysorb—a chemical oxygen scavenger was reported by Khosravitabar and Hippler (2019) in the model organism *Chlamydomonas reinhardtii.* The mechanism behind a 2–5-fold increased bio-hydrogen generation using oxysorb is that the oxygen scavenger induced anoxia in the culture medium without affecting the PSII activity which further facilitated hydrogen production. Genetic manipulation offered promising solutions for enhanced hydrogen accumulation in algal systems (Nyberg et al. 2015). Usage of microRNA (miRNA) to regulate the PSII genes in *Chlamydomonas reinhardtii* have been extensively studied. A blue light inducible system was experimented to activate an artificial miRNA which established an optogenic gene regulation targeting the D1 protein in *Chlamydomonas reinhardtii*. The modified gene overcame the hydrogenase activity setbacks, thereby accelerating the hydrogen accumulation (Wang et al. 2017). A comprehensive review on the emerging approaches in genetic modification for strain development and improved photobiological hydrogen production in microalgae have been studied by Anwar et al. (2019). The model microalgae for biohydrogen, *Chlamydomonas reinhardtii, Nannochloropsis *sp., *Scenedesmus obliquus* are flexible to genetic modification and strain development. This could lead co-production of other forms of bioenergy and other valuable by-products in a refinery platform.

## Latest technologies in algal biorefinery: sustainable routes to biomass valorisation

In recent years algal products have instigated potential ventures for food, medicines, fuels, speciality chemicals, waste management and carbon capture. However, the industry is still in its primitive stages due to the challenges to commercialization which includes its high growth cost, lack of a single harvestation method, strain selection etc., (Awasthi et al. 2020). Despite the bottlnecks in the present scenario, the industry is still booming mainly due to the expanding demand in the global market. Biofuel integrated carbon capture and/or waste treatment would further will not only minimise the cost but also leaves the process with zero waste. Uses of genetic approaches for strain improvisation ensures that the bio-chemical properties of the selected alga remains constant (Bhalamurugan et al. 2018). Production of multiple products from algal cultivation in a biorefinery process is envisaged as as a promising strategy towards commercialisation.

Biorefinery is a sustainable approach to exploiting every component of biological feedstock to develop one or more products or processes in an energy-efficient manner (Koyande et al. 2019b). Microalgal biorefinery is a promising solution for environmental concerns such as atmospheric pollution, fossil fuel depletion coastal pollution etc. These modern technological processes functions towards blue-bioeconomy, by remarkably reducing the cost of algal biomass processing while leading to comprehensive utilisation of the metabolites (González-Delgado and Kafarov 2011). To further enhance the economic viability of the algal biorefinery, closed loop approaches aim at total energy recovery coupled with extraction of biotechnologically viable substances in a food, feed and fine chemical viewpoint (Mohan et al. 2019). Algae are bio-cell factories of intra and extra cellular metabolites and are excellent sources of PUFA, pigments, antioxidants, oleochemicals, polysaccharides, proteins and biofuels. The valorisation and pre-treatment methods of algal biomass, which includes anaerobic digestion, fermentation, thermo-chemical liquefaction, and transesterification, can be integrated or further channelled to bio-processes as bio-remediation, carbon sequestration, bio-methanation etc. for in biorefining platform. The versatility of algae that makes the whole biomass made available unlike the other biological feedstocks signifies in holistic position in bioeconomy framework (Mohan et al. 2019). Table 2 highlights the significance of the advancements in the valorisation technology platform and the widening scopes for algal biorefinery.

**Table 2:** Emerging trends in algal biomass valorisation and biorefinery potentials

No.	Valorisation method	Organism	Target bio-product	Biorefinery approach	References
1	Anaerobic digestion (AD)	*Scenedesmus obliquus* and *Chlorella vulgaris*	A 14% higher biomethane and waste recovery	I. Waste utilisation II. Substrate complementarity	Assemany et al. (2019)
2	Anaerobic digestion	*Saccharina latissimi* and *Fucus serratus*	Enhanced biomethane recovery of 185 mL CH_4_/g VS at 150 °C	Hydrothermal carbonisation integrated AD	Brown et al. (2020)
3	Anaerobic digestion	*Chlorella* and *Phormidium* sp.	A significant 42% biocrude yield and 200 mL/gVS biomethane	Sequential hydro pyrolysis integrated AD	Choudhary et al. (2020)
4	Constant magnetic field applied AD	Cyanoprocayota, Chlorophyta, and Bacillariophyceae	Biogas production 281.1 L of biogas/kg VS with a 41% Biomethane yield	Nil	Dębowski et al. (2016)
5	Hydrothermal treated AD	Microalgal consortia	Biochar and biomethane	Hydrothermal carbonization and anaerobic digestion	Marin-Batista et al. (2019)
6	Anaerobic hybrid reactor	*Scenedesmus obliquus*	Enhanced energy (biomethane) recovery compared to the pre-treated biomass	Nil	Assemany et al. (2018)
7	Anaerobic digestion of residual algal cake	*Nannochloropsis* sp.	48% enhanced methane yield from wet biodiesel extracted biomass	Biodiesel coupled biomethane generation	Kinnunen et al. (2014)
8	Anaerobic digestion	*Chlorella CG12* and *Desmodesmus GS12*	49.87% and 22.26% enhanced biomethane and 10–12% lipid recovery	Biodiesel recovery defatted microalgae by AD	Srivastava et al. (2020)
9	Anaerobic digestion of the lipid extraction biomass by super critical CO_2_ extraction	*Isochrysis* sp.*, Tetraselmis *sp.*, Scenedesmus lmeriensis, Nannochloropsis gaditana*	Enhanced lipid and methane yield—*Tetraselmis* sp. (11% lipid and (236 mL CH4/g VS methane); *S. almeriensis *(10%)	Extraction of different biofuels from algal biomass	Hernández et al. (2014)
10	Biochemical extraction of lipids, proteins and pigments and AD of the residual biomass	*Chlorella vulgaris*	Methane yield were consistent with residual and raw microalgal biomass (207–237 mL CH_4_/g volatile solids)	Value added metabolites and biogas production	Markou et al. (2020)
11	AD of de-oiled biomass and co-digestion with glycerol	*Tetraselmis suecica*	Enhanced methane yield from 173.78 ± 9.57 to 438.46 ± 40.50mL of methane per gram of volatile solids using defatted biomass	Residual biomass used for enhanced biomethane recovery	Santos-Ballardo et al. (2015)
12	Fe2O3 nano catalyst aided transesterification	*Neochloris oleoabundans UTEX 1185*	Enhanced biodiesel (81%) as compared to conventional methods (64%)	Post biodiesel recovery- defatted biomass used for dark-bio-hydrogen production and bio-ethanol by AD along with Saccharomyces cerevisiae (INVSC-1)	Banerjee et al. (2019a)
13	Carbon sequestration and transesterification using a bubble column reaction	*Neochloris oleoabundans UTEX 1185*	Obtained a biodiesel suitable FAME profile and sequestered 1.503 g of CO_2_ from air	Biodiesel and concomitant CO_2_ Sequestration	Banerjee et al. (2019b)
14	Direct transesterification and acid hydrolysis	*Chlorella* sp.	FAME yield—256 g/kg-biomass; coproduction of sugars, proteins and pigments	Zero waste biorefinery approach for biofuel and fine chemicals	Mandik et al. (2020)
15	Carbon capture aided transesterification	*Phormidium valderianum BDU20041*	CO_2_ fixation rate—56.4 mg C/L/d; Lipid content—12.7% and bio calcite removal	Carbon sequestered biomass for biodiesel and bio-calcification	Dineshbabu et al. (2017)
16	Base catalysed transesterification coupled acetone–butanol–ethanol (ABE) fermentation with different pre-treatments	*Chlamydomonas reinhardtii CCAP 11/32 C*	Biobutanol recovery—10.31% g/g cdw; Biodiesel yield—3.82% g/g cdw	Bio-butanol and biodiesel from algal biomass	Figueroa-Torres et al. (2020)
17	Enzyme catalysed direct transesterification followed by ethanol fermentation	*Scenedesmus* sp.	Total FAME yield—92%; bioethanol—86% and glycerol recovery—93%	Algal feedstock for multiproduct recovery	Sivaramakrishnan and Incharoensakdi (2018)
18	Dark fermentation for biohydrogen production	Mixed microalgal consortia	Significant enhancement in biohydrogen yield	De-oiled algal biomass (DAB) for bio-hydrogen and recovery of volatile fatty acids	Subhash and Mohan (2014)
19	Algal fermentation of de-oiled algal biomass (DAB) with hybrid pre-treatment (PT) method	*Chlorella* sp. and* Scenedesmus* sp.	Higher sugar yield in hybrid PT—0.590 g/g DAB Biopolymer PHB—0.43 ± 0.20 g PHB/g DCW	Bioethanol and biopolymer production in a biorefinery framework from DAB	Kumar et al. (2019)
20	Acid and alkali catalysed transesterification coupled anaerobic fermentation	*Padina tetrastromatica*	Recovery rate—7.8% biodiesel and 83.4% bioethanol	Integrated biofuel generation from marine macroalgae	Ashokkumar et al. (2017)
21	Valorisation of industrial waste and industrial flue for algal biomass production	*Chlorella vulgaris*	Enhanced nutrient removal up to 75%; up tp 5% CO_2_ captured, elevated lipid accumulation up to 34%	Waste mitigation coupled carbon sequestration	Yadav et al. (2019)
22	Individual valorisation of algal metabolites	*Sargassum muticum–Brown algae*	I. Supercritical fluid extraction with CO_2_—bioethanol generation II. Hydrothermal processing—for extraction of fucoidan and phlorotannin compounds	Various valorisation process for extraction of viable biotechnological products in a biorefinery viewpoint	Balboa et al. (2015)
23	Waste treatment and lipid production	*Ascochloris* sp.* ADW007*	I. Up to 80% clean odourless water with reduced COD obtained II. Enhanced lipid productivity	Bioremediation of dairy waste and lipid recovery in a biodiesel viewpoint	Kumar et al. (2019)
24	Effluent treatment of Palm Oil Mill effluent and biomass production for industrial applications	*Scenedesmus* sp.* and Chlorella* sp.	I. Integrated system showed pronounced nutrient uptake and carbon capture II. Lipid profile ideal for industrial usage	Bioremediation, carbon capture and exploitation of biomass—closed loop approach	Hariz et al. (2019)
25	Enhanced protein recovery using pulse electric field cyclic protein extraction	*Chlorella vulgaris*	Extracted free protein during cultivation—96.6 ± 4.8%	A closed loop-biorefinery approach by continuous extraction of protein in the cultivation phase	Buchmann et al. (2019)

Algal biofuels are established channels to develop biorefinery and have gained industrial momentum in the recent years. Co-production of other biofuels has been explored using the model organisms *Chlamydomonas reinhardtii* and *Scenedesmus obliquus* in a biorefinery perspective as they hold strong literature values. The unique carbon concentrating mechanisms of the microalgae come in play when grown in a flue gas environment. Singh et al. (2014) investigated carbon recycling and biodiesel recovery from an endolithic cyanobacterium *Leptolyngbya* sp., which successfully created biorefinery venues. With proven results in laboratory platforms for biorefining, a novel industrial site which coupled carbon sequestration of industrial flue gas and biocalcification was demonstrated by Dinesh babu et al. (2017). Cyanobacterium *Phormidium *sp., revealed growth for 10 days in un-scrubbed flue gas and passed out with a pronounced CO_2_ fixation rate of 56.4 mg C/L/d. While FAME profile of the carbon captured cyanobacterial biomass proved to be an ideal candidate for biodiesel with presence of favourable fatty acid profile (C14–C18). A number of such emerging trends in algal biorefinery including anaerobic digestion combined bioremediation, bioenergy integrated nutrient removal, waste treatment, carbon sequestration was critically reviewed (Veerabadhran et al. 2021; Milledge et al. 2019). However, the key driver behind a successful algal biorefinery model is the strain identification and speciation via extensive literature study, molecular characterisation and high throughput screening methods to identify the potent alga with intrinsic commercial value and appreciable productivities. A profound understanding of the competent strain and its built-in biomass dynamics, compatibility to optimisation and multiple processing routes are inevitable for biorefining and cost management of algal bio-processes. Fractional pathways and combined algal processing (CAP) are competent technologies that empowers biomass valorisation in developed or modified strains (Laurens et al. 2017). Fractionation of biomass can bring upon multiple channels for biorefinery based on biofuels and essential metabolites such as the oleochemicals, polymers, bioplastics, surfactants, etc. While, combined algal biomass processing aims at extracting a specific product without compromising the quality and quantity of the co-metabolites thereby leaving the residual biomass for further processing (Dong et al. 2016). The existing investigations on algal biorefinery suggest that algae are self-sustainable microbial cell factories and could promote mankind a step closer towards blue-bioeconomy.

### Integration of an algal biorefinery to the circular economy

The metabolic and genetic versatility of algae enables downstream processing of a single product to a multi-product biorefinery in a closed loop modus, which further enhances the refining process’s environmental sustainability and economic viability (Mohan et al. 2019). Biorefining in algal systems can be hastened by integrating the pre-treatment and extraction methods with the state-of-the-art technologies like integrated downstream processing, integrated multi-product extraction, combined algal biomass processing etc. (Vermuë et al. 2018). A positive bio-economy model using algae is further strengthened by techno-economical assessments and Life Cycle Assessments (LCA) to produce bulk products at a reduced cost pro-rata to the market size (Ruiz et al. 2016). Techno-economic assessments and pilot scale data generation are indispensible for industrial microalgal biorefinery processes as production of targeted products are not economically viable and technologically sound (Subash et al. 2022). Microalgae *Chlorella *sp. and *Scenedesmus* sp. were employed in numerous closed-loop biorefinery models (Mohan et al. 2019). Resource-circular algal biorefining was achieved by integrating biofuel, bio-fertiliser and biogas co-production from microalgae cultivation in sugarcane waste and the AD of residual algal biomass. Process integration notably upsurged the annual production rate of algal biofuel and biogas while cutting down the production costs (Zewdie and Ali 2020).

Waste utilisation and algal biomass valorisation is considered promising to attain value added metabolites in an energy efficient manner. Avila et al. (2022) evaluated mono and co-digestion methods to remediate winery waste in a circular bioeconomy perspective. Comprehensive waste utilisation and minimisation can be achieved in co-digestion methods where the treated water and the dried residual contents can be used for irrigation and as plant fertilizer respectively. Mixotrophic cultivation of high lipid yielding algae in waste streams is a plausible course to circular economy via carbon capture (Arun et al. 2020). Yin et al. (2018) demonstrated DHA production from *Schizochytrium *sp. cultured in fermentative and microbial waste. The method minimised waste and upsurged the lipid (63.63 g/L) and DHA (28.45 g/L) yields. A seaweed biorefinery investigation conducted by an Irish macroalgal processing plant revealed augmented scope for circular economy approach with appreciable yields for biogas and biotechnology viable metabolites from *Laminaria* sp. and *Fucus *sp. (Tedesco and Stokes 2017). The key factor for success of blue-bioeconomy is sustainability, which indeed relies on novel biological feedstocks and the advanced valorisation methods looped into biorefinery by effective integration of the product recovery and waste management (Fig. 2). Algae are definitely an untapped source for viable products however it demands the intersection of technology and academia to achieve eco-friendly and superior products.

## Future trends and applications in potential food/agriculture/pharma fields

Algae are promising feedstocks for commercial production of viable metabolites that could potentially replace synthetic food and feed supplements. These viable compounds possess wide applications over an array of agricultural, pharmaceutical, plastics and polymers, cosmeceuticals, nutraceuticals and oleochemical industries. High value products from microalgae are markedly used as pharmaceutical compounds. β-Carotene, PUFA and zeaxanthin hold significant value in pharmaceutical industry. β-Carotene from algal biomass are an emerging vitamin-A supplement, besides being used as ‘anti-cancer agents’(Bhalamurugan et al. 2018). Cyanovirin produced by *Nostoc* sp., is globally used to treat HIV sumptoms and are used as a potent anti-viral agent (Bhattacharjee et al. 2016). High value proteins namely the β-insulin, IgA, erythropoietin extracted from *Chlamydomonas reinhardtii* are cultured for production of pharmaceutical proteins. *Chlorella* and *Chlamydomonas* are the largely used algae for pharmaceutical purposes (Santhosh et al. 2016). Modern algal biomass valorisation methodologies have fastened the way out for global algal industries to produce sustainable, environment friendly, zero waste, cost effective products. Literally, algal biomass can venture into various agricultural and biotechnological industries that manufacture viable products for humans and other living forms. Their state in the global energy market is growing every year which shows immense potentials for green technologies (Piwowar 2020). In recent years, advanced technologies namely the synthetic biology, combined algal biomass processing, phenomics and Industry 4.0 approach etc., play phenomenal roles in improvising the algal metabolites (Fabris et al. 2020). Industry 4.0 applies machine to machine, plug and play technologies in what is called as “Internet of Things (IoT)” approach (Tao et al. 2018). These advanced methods are called the “digital twin” and makes simulations for the real time data trajectories such that the demand and the yield match exactly and leaves no cost on over production of target compounds (Uhelmann et al. 2017). Despite being highly flexible to genetic modifications, and versatile in its habitat distribution, the practicality of utilising algal biomass in an industrial perspective relies on its processing technology on a green platform with an appreciable scale transfer ration. Additionally, global production of algal products needs to be supported by universal regulation or standards that governs the commercial production of algal metabolites for various applications (Bernal et al. 2011).

The feedstock potentials of algal biomass and its vast applications have been discussed in the earlier sections of this article. List of industrial made algal products as dietary supplements, nutraceutical and therapeutical compounds are presented in Table 2. With newer technologies such as use of hydrothermal systems, accelerates the scope of algal biorefinery and supports superlative product recovery (Morales-Contreras et al. 2022). With the present rate at which algal industry is growing, it is expected to channel positively to attain blue bio-economy backed up by its land free cost-effective cultivation methods (Ullmann and Grimm 2021). The way ahead has purely relied on algal biorefining and continuous research and development activities by national and internationally funded research programs, which would in turn transform the laboratory technologies to land for sustainable algal products.

### Global market view of the value added products from algae

Global demand for algal products is growing rapidly owing to the vast and varied benefits of the algal metabolites. Presently, the microalgal market is fragmented, as the industry has recently gained momentum and is undergoing periodic transition in terms of collaboration, newer technologies, addition of novel products etc., The global demand for algal products is mainly attributed to health and dietary products, pharma and neutraceutical industry and biofuels in recent years. According to published reports of “Global forecast to 2028”, the market value for microalgal products is expected to reach a maximum of 1.8 billion by the year 2028. The CAGR growth from 2021 is projected to be 10.3% (Meticulous research®, https://www.globenewswire.com/en/news-release/2021/09/13/2295918/0/en/Microalgae-Market-is-Expected-to-Reach-1-8-Billion-by-2028-Market-Size-Share-Forecasts-Trends-Analysis-Report-with-COVID-19-Impact-by-Meticulous-Research.html). Of the several industrially significant algal species that research has identified, *Spirulina* holds the major contribution in the global market owed to its immense health benefits to mankind and is so far the predominantly used species in aquaculture industry. An article published in Business insider (2014), acknowledged algae as the super food and these slimy organisms are expected to be the major contributor in the global market (health, pharma, cosma and neutraceutical industry) by the year 2025 and would instigate the largest movement towards “circular- bioeconomy”.

## Conclusions

With the exalting civilisation, mankind has realised the significance of products obtained from natural feedstocks. When renewability becomes a crucial factor for the commercial production of food, feed and fuel, algal biomass holds a prominent standpoint in the global market. This comprehensive review conceptualises the emerging trends and the state of art technologies in the algal biomass valorisation for novel biotechnological products. We have attempted to enlist the latest technological advancements, novel valorisation methods that has taken the algal biorefinery framework a step closer toward the blue-bioeconomy. The key steps involved in enhancing the efficacy of biomass valorisation are (a) understanding of the algal strains by biochemical and synthetic methods to its true potentials and novelties, (b) simplified pre-treatments, integrated extraction and downstream processing (c) one step conversion technologies for maximum valorisation. We envision that merging of the existing valorisation methods with the newer technologies which could enhance the market scope of the algal products with reduced cost and enhanced sustainability. The economics of the algal biorefining could be accelerated by integrated algal processing and closed circular approaches that would bring in “zero waste”, “carbon neutral” algal technologies. A sustainable circular bioeconomy will effectively replace the synthetic metabolites and nutraceuticals and could overcome the deficits of the land-based crops when it comes to renewability. Above all, algae can offer promising solutions to environmental concerns by producing clean zero sulphur, carbon neutral renewable fuels, while successively capable of degrading complex waste streams and mitigating climate change by sequestering atmospheric CO_2_ and industrial CO_2_ emissions. Research and development in algal valorisation methods would take the trajectory towards complete harvesting of the rich biochemical pool of algae. Future research in algal biomass valorisation need to be focused on diminishing the capital burden of cultivating algae and waste recycling. Holistic extraction of value added metabolites and utilisation of complex waste loadings and gaseous substances is imperative. This review systematically collates the latest trends on algal biomass valorisation and the plausible approaches to overcome the bottlenecks in algal biorefineries.
